# Sugars, obesity, and cardiovascular disease: results from recent randomized control trials

**DOI:** 10.1007/s00394-016-1257-2

**Published:** 2016-07-14

**Authors:** James M. Rippe, Theodore J. Angelopoulos

**Affiliations:** 1 Rippe Lifestyle Institute, 21 North Quinsigamond Avenue, Shrewsbury, MA 01545 USA; 2 School of Health Sciences, Emory and Henry College, Emory, VA 24327 USA

**Keywords:** Sugars, High-fructose corn syrup, Sucrose, Obesity, Cardiovascular disease

## Abstract

The relationship between sugar consumption and various health-related sequelas is controversial. Some investigators have argued that excessive sugar consumption is associated with increased risk of obesity, coronary heart disease, diabetes (T2D), metabolic syndrome, non-alcoholic fatty liver disease, and stimulation of reward pathways in the brain potentially causing excessive caloric consumption. These concerns have influenced organizations such as the World Health Organization, the Scientific Advisory Committee on Nutrition in England not to exceed 5 % of total energy and the Dietary Guidelines for Americans Advisory Committee 2015 to recommend upper limits of sugar consumption not to exceed 10 % of calories. Data from many randomized control trials (RCTs) do not support linkages between sugar consumption at normal levels within the human diet and various adverse metabolic and health-related effects. Fructose and glucose are typically consumed together in roughly equal proportions from high-fructose corn syrup (also known as isoglucose in Europe) or sucrose. The purpose of this review is to present data from recent RCTs and findings from recent systematic reviews and meta-analyses related to sugar consumption and its putative health effects. This review evaluates findings from recent randomized controlled trials, systematic reviews and meta-analyses into the relationship of sugar consumption and a range of health-related issues including energy-regulating hormones, obesity, cardiovascular disease, diabetes, and accumulation of liver fat and neurologic responses. Data from these sources do not support linkages between sugar consumption at normal levels within the human diet and various adverse metabolic and health-related effects.

The world is in the midst of twin epidemics of obesity and diabetes often lumped together as diabesity (T2D) [1–6]. It has been estimated that there are over one billion individuals worldwide who are currently obese [1]. This number is anticipated to rise to 1.5 billion individuals by the year 2030 if current trends continue [1]. In the USA, it has been estimated that 33.8 % of adults, over 66 million individuals, are obese, while an additional 74 million are overweight [7]. The prevalence of obesity grew a shocking 40 % over the last 30 years [8]. The obesity epidemic is truly global. In European countries, obesity ranges from 20 to 30 % and is even higher in Australia, South America, Middle-East, and Polynesia [1].

The International Diabetes Federation estimates that there are over 300 million individuals currently living with diabetes [5]. This number is predicted to double by the year 2035 [6, 7]. Heart disease remains the leading cause of mortality worldwide and accounts for over 37 % of all mortality in the USA on an annual basis [9]. Non-alcoholic fatty liver disease (NAFLD) now represents the leading cause of liver failure and the need for liver transplantation worldwide [10, 11]. Multiple studies have now showed that these metabolic conditions are inter-related.

With these grim statistics in mind, it is not surprising that investigators have looked at various aspects of nutrition to delineate potential underlying associations and causes and recommend possible ways of ameliorating these inter-related metabolic conditions. Recent attention has been focused on the consumption of sugars, in general, and fructose containing sugars in particular [12, 13]. Some epidemiologic studies have linked consumption of added sugars to increased risk of weight gain and obesity [14–16], increased risk factors for cardiovascular disease (CVD) [17] including hypertension [18–20] and dyslipidemia [21, 22], and increased risk factors for diabetes [23–25].

Potential linkages between added sugars and various diseases have not only public health implications, but also impact on public policy. The World Health Organization (WHO) has issued guidelines recommending that added sugars should constitute no more than 5 % of overall calorie consumption [26]. The Scientific Advisory Committee on Nutrition in England (SACN) [27] has also recommended an upper limit not to exceed 5 %, while the 2015 Dietary Guidelines Advisory Committee (2015 DGAC) in the USA has recommended an upper limit not to exceed 10 % of calories [28]. The Food and Drug Administration in the USA has followed the lead of the 2015 DGAC and recommended an upper limit of no more than 10 % of calories from added sugars [29]. In contrast, the European Food Safety Authority (EFSA) [30] found no harm in fructose consumption up to 25 % of total calories which is also consistent with the Institute of Medicine (IOM) carbohydrate report which found no harm in fructose containing sugars up to 25 % of calories [31].

In this contentious and controversial debate, it is important that members of the scientific community have to stand on firm ground and base opinions on the best available science. The purpose of the current review is to provide evidence from recent randomized controlled trials conducted in our research laboratory and others as well as findings from recent systematic reviews and meta-analyses related to sugar consumption and its putative health effects. We conclude that high quality evidence does not support a unique link between added sugars and various adverse health effects when consumed at normal levels, particularly in isocaloric settings.

## Theoretical concerns

It has been known for many years that fructose and glucose are handled differently in the human body. Ninety percent of fructose is metabolized in the liver on first pass [32]. In contrast, glucose can be metabolized in multiple cells throughout the human body. Both fructose and glucose are transported into hepatocytes utilizing the facilitator glucose transporter GLUT2. Once inside the hepatic cells, however, the pathways for metabolism for fructose and glucose are considerably different.

First, fructose does not increase glycemia in contrast to glucose and, hence, does not stimulate insulin release. Fructose is phosphorylated into fructose-1-phosphate with phosphate donated by ATP which is metabolized into ADP and ultimately to AMP and finally to uric acid. The accumulation of uric acid has been argued to play a role in the development of high blood pressure [33]. This will be discussed in more detail in subsequent sections of this review. Fructose is phosphorylated by the enzyme fructokinase and subsequently cleaved into the triokinase phosphorylates glyceraldehyde to glyceraldehyde phosphate. It is important to note that at the level of glyceraldehyde phosphate, the metabolism of fructose and glucose metabolism is interactive [32]. Fructose cannot be utilized for cells in the body, and thus, there are pathways to convert it into glucose and other metabolizable molecules. Over half of fructose is converted to glucose, another 15 % to glycogen, 15–20 % to lactate, and a few percent to carbon dioxide [32]. This has been demonstrated in multiple tracer studies as summarized by Sun and Empie [34]. A small percentage of ingested fructose can ultimately be converted into free fatty acids which are ultimately packaged into triglycerides (TG) and carried into the blood stream complexed with very low-density lipoprotein (VLDL). The amount of fatty acids that are produced through this mechanism called de novo lipogenesis has been estimated in elegant studies by Hellerstein et al. [35] to be on the order of 1–5 % of ingested fructose even under settings of extreme overfeeding. The amount of fat produced though this process is in the order of one gram per day compared to the 100–125 g typically consumed each day in the human diet. Nonetheless, some investigators have argued that de novo lipogenesis is an important pathway leading from fructose consumption to fatty liver and insulin resistance.

## Energy-regulating hormones

The differences in metabolism between fructose and glucose in humans have led a number of investigators to explore the impact of these two monosaccharides on energy-regulating hormones including insulin, leptin, and ghrelin [36, 37]. Teff et al. [36] reported that consuming either fructose sweetened beverages or glucose sweetened beverages at the level of 25 % of calories was resulted in lowering of 24 h concentrations of circulating insulin, glucose, and leptin and decreased postprandial suppression of plasma ghrelin concentrations. Other investigators have utilized a similar model in overweight or obese subjects and demonstrated that fructose sweetened beverages, when compared to glucose sweetened beverages, when consumed at 25 % of energy, were resulted in increased postprandial triglyceride concentrations in overweight/obese subjects compared to normal weight subjects [37]. These effects were more pronounced in men than women. It should be noted that in this model, the sugars are given as monosaccharides—namely, either glucose or fructose— despite the fact that neither of these monosaccharides is consumed in isolation to any appreciable degree in the human diet. This has created confusion since a number of scientists have speculated that these observed differences in energy-regulating hormones could create an environment resulting in increased hunger, food consumption, and obesity when consuming fructose [38, 39].

The differences between fructose and glucose effects on energy-regulating hormones have also led to further speculation that high-fructose corn syrup (HFCS) might be more obesogenic than sucrose. This speculation was based on the misperception that HFCS contains considerable higher amounts of fructose when compared to sucrose. The proper comparison, however, is HFCS (isoglucose) versus sucrose since both contain approximately 50 % of fructose and 50 % of glucose. (HFCS-55, which is the most commonly utilized form of HFCS in beverages, contains 55 % of fructose, while HFCS-42, which is the most commonly utilized form of HFCS in baked goods and other applications, contains 42 % fructose [40]. The rest of both of these forms of HFCS is glucose or glucose polymers).

Our research laboratory has conducted four trials looking at the effects of either HFCS vs sucrose or HFCS vs sucrose, glucose or fructose on energy-regulating hormones. In the trial published by Melanson et al. [41], we demonstrated that there we no different acute impacts (24 h) when comparing 25 % of calories either from HFCS or sucrose on insulin, glucose, leptin, or ghrelin in non-smoking women 20–60 years of age with body mass indexes of 19–25 kg/m^2^. Subsequent research trials in our laboratory evaluated energy-regulating hormones in obese individuals and found no differences between HFCS and sucrose [42].

A third study in our laboratory showed that there were no differences when comparing 8 % of calories from either HFCS or sucrose (25th percentile population consumption level) to 18 % of calories from these two sugars (50th population level in the United States) and 30 % of calories from these two sugars (90th percentile population consumption level) over a 10 week period [43]. We have subsequently shown that there are no differences in energy-regulating hormone responses when comparing average levels of consumption of fructose (9 % of calories to glucose, 9 % of calories from glucose, 18 % of calories from HFCS or sucrose), (unpublished data). Thus, assertions that metabolic differences between fructose and glucose can lead to an environment that promotes weight gain and obesity must be treated with extreme caution particularly when these two monosaccharides are evaluated at levels far beyond the normal human consumption level and in isolation in contrast to how they are normally consumed in the human diet which is invariably together.

## Sugars and obesity

The modern concern about a potential role of sugars as a unique cause for obesity seems to have originated with a review article published in the *American Journal of Clinical Nutrition* in 2004 by Bray, Nielsen and Popkin [39]. This article raised the provocative question of whether there was a unique relationship between HFCS consumption and the rapid increase in the obesity epidemic in the USA. These researchers noted a temporal relationship between increasing consumption of HFCS and obesity and argued that the metabolism of fructose could result in increased likelihood of overconsumption of calories leading to obesity and associated metabolic diseases including CVD, T2D, and metabolic syndrome (MetS).

Multiple subsequent studies from research laboratories have not demonstrated any unique properties of HFCS compared to sucrose with regard to energy-regulating hormones, appetite or weight gain both in normal weight and obese individuals [41, 43–45]. Both the Academy of the Nutrition and Dietetics [46] and the American Medical Association [47] have issued statements concluding that there are no differences between HFCS and sucrose with regard to the likelihood of causing obesity.

The literature related to sugars and obesity has subsequently shifted to a consideration of whether any fructose containing sugars such as sucrose, HFCS, or concentrated fruit juices may contribute to obesity. Several meta-analyses have suggested that sugar sweetened soft drinks (SSBs) are associated with weight gain and obesity in both children and adults [14, 15]. There have been three recent systematic reviews and meta-analyses of RCTs on sugar consumption or SSB consumption and body weight [48–50]. These trials have suggested that increasing energy consumption by increasing sugar intake in adults may lead to modest weight gain; however, the weight gain appears to be due to the increased energy consumption rather than any unique aspect of sugars per se.

The systematic review and meta-analysis by Te Morenga et al. [49] included 30 randomized controlled trials (19 of which were ad libitum and 11 were isoenergetic) and 38 prospective cohort studies. The “ad libitum” randomized controlled trials and cohort studies agreed with each other that when individuals either reduced or increased sugar consumption a “small but significant” effect on body weight occurred. On average, individuals in the ad libitum trials and cohorts studies lost 0.8 kg and when they reduced their sugar intake, and gained an average of 0.75 kg when they increased their sugar consumption. The 11 isocaloric trials, however, showed no association between body weight change and sugar intake. This caused these investigators to conclude “the most obvious mechanism by which increased sugars might promote weight gain is by increasing energy consumption to an extent that exceeds energy output and restores energy balance… we observed that isoenergetic replacement of dietary sugars with other macronutrients resulted in no change in weight. This finding strongly suggested that energy balance is a major determinant of the potential for dietary sugars to influence measures of body fatness… the data suggests that the change in body fatness that occurs from modifying intake of sugars results from in alteration in energy balance rather than physiologic or metabolic consequence of monosaccharides or disaccharides.” [49].

It should also be noted that between 1970 and 2010 in the USA average total energy intake increased by 474 calories per person [51]. Virtually all of this increase in energy intake (approximately 94 %) can be attributed to an increase in flour and cereal products and added fats, while added sugars only contributed 7 % of the total increased caloric intake. Thus, data related to added sugar intake as a potential significant contributor to weight gain and obesity must be treated with great caution. Moreover, public policy attempts to limit sugar consumption as a mechanism for helping individuals control weight seem unlikely to succeed.

## Sugars and cardiovascular disease

There are, to our knowledge and for obvious reasons, no reported RCTs examining the effect of sugar consumption on CHD itself. Three prospective cohort studies have examined the association between SSB consumption and incident of CHD [52–54]. Eshak et al. [52] reported a large prospective cohort study and found no association between SSB consumption and myocardial infarction. de Koning et al. [53] reported data from the Male Health Professional Follow-up Study and found a significant association between CHD events and the highest quintile of SSB consumption compared to the lowest. Fung et al. [54] reported data from the Nurses’ Health Study and found significant elevated risks associated with CHD comparing greater than or equal to two servings of SSB per day compared to <1 serving per month. Te Morenga and colleagues reported a systematic review and meta-analysis of 12 trials most of which had high levels of sugar consumption—typically >95th percentile population consumption [55]. These investigators reported no increase in systolic blood pressure and a small increase in diastolic blood pressure although the absolute value of increase was low (1.4 mmHg).

Most RCTs exploring sugars and CHD have focused on risk factors for CHD rather than CHD itself.

### Blood pressure

Several RCTs have examined whether fructose itself or fructose containing sugars contribute to increased blood pressure. Brown et al. [56] administered 15 subjects an acute load of fructose (60 g in 500 mL of water), glucose, or pure water and found a significant increase (approximately 3 mmHg) over the 120 min of the study following fructose consumption compared with either glucose or water. Raben et al. [57] randomly assigned 21 overweight individuals to supplements of either sucrose or artificial sweeteners for 10 weeks. Blood pressure increased more in the sucrose group than the group consuming artificial sweeteners. These data, however, are confounded by the fact that these individuals also gained on an average 2.6 kg more than controls.

Other studies have not found increases in blood pressure related to fructose administration. These include studies by Le et al. [58], Maersk et al. [59], and studies from our research laboratory at levels up to the 90th percentile population consumption level of fructose which have not shown increases in blood pressure [60–62]. Moreover, Ha et al. [63] performed a meta-analysis of 13 randomized and non-randomized controlled feeding trials where subjects were given an isocaloric exchange of fructose for carbohydrates and found no increases in blood pressure.

### Lipids

A number of studies have explored the potential linkage between consumption of added sugars and dyslipidemia [55, 64, 65]. As a result of these studies, the American Heart Association recommended restricting consumption of fructose containing sugars as a mechanism for controlling triglycerides (TGs) [66]. Data to support this, however, are inconclusive [67–69]. In isocaloric trials, even large doses of fructose containing sugars have been reported to not show lipid abnormalities [70]. In hypercaloric trials, however, in which fructose was supplemented to background diets thereby creating excess energy, increases in LDL cholesterol and triglycerides have been reported [67–69]. In RCTs in our laboratory individuals who consumed either sucrose or HFCS at 10 or 20 % of calories in an isocaloric diet over 10 weeks in a free living trial, no increases in total cholesterol, TGs, or LDL cholesterol [71] occurred. In a mildly hypercaloric trial, however, a 10 % increase in TGs occurred driven largely by individuals who consumed 30 % of those calories from either HFCS or sucrose [60, 61].

Despite the conflicting data in this area, the American Heart Association has issued guidelines recommending that males consume no more than 150 kcals each day of added sugars and females no more than 100 kcals [72]. (These recommendations limit sugars to 5–7 % of total calories). The 2015 DGAC [28] recommended the upper limit of calories from added sugars to not exceed 10 % citing evidence for increased risk of cardiovascular disease associated with added sugar consumption as “moderate.” Given the mixed nature of RCTs and meta-analyses in this area, however, it appears that these recommendations should be taken with considerable caution.

## Sugars and diabetes

To our knowledge, there are no RCTs examining the effect of sugars on diabetes. Therefore, studies that have been relied upon to suggest a linkage between sugars and diabetes are either lower forms of evidence, (e.g., epidemiologic and ecological studies) or studies which explore risk factors for diabetes.

Several recent ecological analyses have reported that the prevalence of diabetes is correlated with sugar consumption in various countries [24, 25]. Ecological analyses, however, are considered to be a weak form of evidence. Furthermore, as pointed out by Van Buul et al. [73] in one of these studies, in European countries, the authors confused sugar consumption with sugar production. In European Union countries, commodities such as sugar flow freely across borders thus production is very different from consumption. Furthermore, other ecologic analyses have shown markedly different results. For example, in Australia, a 10 % decrease in the contribution of sugar in beverages has recently occurred despite increases in obesity and diabetes [74]. This has been labeled the “Australian Paradox.” Similar “paradoxes” have been seen in the USA [75] and Canada where a 15 % sugar consumption decline has occurred over the past decade, while both obesity and diabetes have increased.

Prospective cohort studies provide mixed evidence concerning sugar consumption and diabetes [16, 76]. Malik et al. [16] reported a meta-analysis of cohort studies related to SSBs and incident diabetes. Of the eight studies these investigators reported, four did not find a significant effect of SSBs on the incidence of diabetes and five did not adjust findings for energy intake and body weight. Moreover, another study published by the same group did not show a relationship between sugar consumption and diabetes risk [76]. A study reported from the US Professionals Health Study Follow-Up cohort showed no association between SSB consumption and diabetes once data were adjusted for total energy intake [77].

Meta-analyses also have generated few data to support an association between sugar intake and diabetes [78–80]. Cozma et al. [81] reported a systematic review and meta-analysis of RCTs and non-randomized controlled trials of fructose and diabetes and reported no adverse impact on glycemic control. Most randomized controlled studies in non-diabetic patients where sucrose or fructose have been substituted in a control diet have not shown adverse effects of multiple risk factors for diabetes [82–86].

Insulin resistance is an established risk factor with diabetes often preceding T2D by 10–20 years [87]. A recent research trial reported by our laboratory compared three fructose containing sugars (fructose itself, HFCS and sucrose), to glucose and unsweetened controls at average consumption levels for fructose [88]. This study demonstrated that added sugar consumed at median American intake levels did not produce changes in measures of insulin sensitivity or glucose tolerance and that no sugar had more deleterious effects than others.

Another RCT from our research group looked at total body insulin sensitivity and hepatic insulin sensitivity using the Matsuda Index and found no increases in either variable after 10 weeks of consumption of average amounts of fructose containing sugars compared to glucose [89]. A report by Johnston et al. [90] showed that overweight men with increased abdominal girth had no increase in either hepatic or total body insulin resistance when studied in a weight stable environment. Nor were there any differences between fructose and glucose when delivered at 25 % of calories. In a second portion of this study, where these same individuals were overfed and gained weight, increases in liver fat occurred. There were, however, no differences between fructose and glucose with regard to this parameter.

Thus, when the totality of high quality evidence is taken into account, there is little support for a relationship between sugar consumption and diabetes. In hypercaloric trials, however, there does seem to be some signal for harm—more likely due to added calories rather than some unique property of sugar per se.

## Sugar and liver fat accumulation

As already indicated, NAFLD has been steadily increasing worldwide over the past 20 years and represents the leading cause of chronic liver failure and the need for liver transplantation [10, 11].

It has been argued that fructose containing sugars may contribute to NAFLD though the process of de novo lipogenesis [33]. However, as already discussed, the amount of fatty acids generated through the metabolism of fructose is relatively small—on the order of 1—5 % of fructose consumed and approximately 1 % in quantity when compared to ingested fats [34, 35].

Several investigators have suggested that large doses of fructose may contribute to ectopic fat deposition in the liver [37, 38, 91–93]. Stanhope et al. [37] reported that individuals who were given 25 % of their caloric intake as fructose, showed increased liver fat after fructose consumption. Le et al. [94] gave descendants of diabetics 3.5 g/kg lean body weight of fructose and found some increased accumulation of liver fat. It should be pointed out that both of these studies used dosages far in excess of normal amounts of consumption of fructose and both used fructose by itself which is normally not consumed in isolation in the human diet. Maersk et al. [66] reported increases in liver fat in individuals who consumed a one liter serving of sugar sweetened beverage compared to diet beverage, milk, and water over a 6 month period. These findings, however, were confounded by weight gain in the cohort.

Other investigators have not found increases in liver fat in short-term studies utilizing either fructose or sucrose. A RCT from our research laboratory which compared either 8, 18, or 30 % of calories from HFCS or sucrose did not find any accumulation in liver fat over a 10 week, free living trial [95]. Thus, there seems to be little evidence for a unique effect of fructose containing sugars leading to NAFLD in typical amounts and ways normally consumed by humans.

## Sugars and neurologic responses

Some animal experiments have suggested differences in brain responses to fructose compared to glucose [96, 97]. However, these experiments must be treated with great caution since animal brains (particularly rodents, which were utilizing many of these studies) differ in numerous significant ways from the human brain.

Recent studies in humans have utilized functional MRI (fMRI) to look at potential different neurologic responses to various sugars in human beings [98–100]. Page et al. [99] compared a 75 g oral bolus of fructose to 75 g oral bolus of glucose in 20 young, healthy volunteers and reported differences in hypothalamic blood flow with glucose suppressing this blood flow and fructose not suppressing blood flow when assessed by arterial spin labeling. Purnell et al. [100] explored neurologic responses to 25 g of either fructose or glucose delivered as intravenous boluses. Blood flow to hypothalamus was not different between fructose and glucose but there were reported differences in blood flow to the cerebral cortex. Both of these studies should be viewed with considerable caution since both utilized monosaccharides rarely consumed in isolation in human beings and, in the instance of Purnell et al., utilized an atypical delivery route (intravenous). Unfortunately, these preliminary experiments have lead some scientists to speculate that fructose containing sugars may lead to a form of “addiction” although the scientific basis such speculation remains very much in doubt [101–103]. A study conducted in our research laboratory examined HFCS or sucrose given at 18 % of calories compared to 9 % of fructose or glucose in low-fat milk compared to unsweetened milk in the context of mixed nutrient meals and showed no differences in hypothalamic blood flow [104].

## Summary and conclusions

Multiple RCTs, as well as recent systematic reviews meta-analyses, have suggested that when sugars are substituted isocalorically for other carbohydrates and consumed in the normal range of human consumption there is nothing unique in regard to sugar consumption and health consequences. Questions remain from research trials where fructose containing sugars have been substituted in ad libitum or hypercaloric research designs. Further studies will be necessary to settle issues in this area.

For now, we believe it is safe to state that the current literature provides little support for a unique relationship between consumption of fructose containing sugars, risk factors, cardiovascular disease, diabetes, or NAFLD at normally consumed amounts in the normal ways that added sugars are typically consumed in the human diet (e.g., sucrose or HFCS). It is important to point out that, however, abundant evidence exists suggesting that consumption of all energy dense nutrients, including added sugar, represents an important step along with decreased physical activity in increasing the risk of interrelated metabolic diseases such as obesity, CHD, T2D, and NAFLD.

## References

[1] WHO, FAO (2003). Expert consultation on diet, nutrition and the prevention of chronic diseases: report of the joint WHO/FAO expert consultation.

[2] Monteiro CA, Moura EC, Conde WL, Popkin BM (2004). Socioeconomic status and obesity in adult populations of developing countries: a review. Bull World Health Organ.

[3] Monteiro CA, Conde WL, Lu B, Popkin BM (2004). Obesity and inequities in health in the developing world. Int J Obes Relat Metab Disord.

[4] Popkin BM (2006). Global nutrition dynamics: the world is shifting rapidly toward a diet linked with noncommunicable diseases. Am J Clin Nutr.

[5] International Diabetes Federation (2011) Global burden. IDF Diabetes Atlas, 6th edn. http://www.idf.org/sites/default/files/EN_6E_Ch2_the_Global_Burden.pdf

[6] International Diabetes Federation (2012) IDF Diabetes Atlas. http://www.idf.org/diabetesatlas/5e/Update2012

[7] Flegal KM, Carroll MD, Ogden CL, Curtin LR (2010). Prevalence and trends in obesity among US adults, 1999–2008. JAMA.

[8] Center for Disease Control and Prevention (CDC) (2010) Overweight and obesity: US obesity trends. U.S. Department of Health and Human Services, Centers for Disease Control and Prevention, Atlanta. http://www.cdc.gov/obesity/data/trends.html. Accessed 9 Dec 2015

[9] Rosamond W, Flegal K, Friday G, Furie K, Go A, Greenlund K, Haase N, Ho M, Howard V (2007). American Heart Association Statistics Committee and Stroke Statistics Subcommittee. Heart disease and stroke statistics—2007 update: a report from the American Heart Association Statistics Committee and Stroke Statistics Subcommittee. Circulation.

[10] Clark JM (2006). The epidemiology of nonalcoholic fatty liver disease in adults. J Clin Gastroenterol.

[11] McCullough AJ (2002). Update on nonalcoholic fatty liver disease. J Clin Gastroenterol.

[12] Bray GA, Popkin BM (2014). Dietary sugar and body weight: have we reached a crisis in the epidemic of obesity and diabetes? Health be damned! Pour on the sugar. Diabetes Care.

[13] Kahn R, Sievenpiper JL (2014). Dietary sugar and body weight: have we reached a crisis in the epidemic of obesity and diabetes? We have, but the pox on sugar is overwrought and overworked. Diabetes Care.

[14] Olsen NJ, Heitmann BL (2009). Intake of calorically sweetened beverages and obesity. Obes Rev.

[15] Malik VS, Schulze MB, Hu FB (2006). Intake of sugar-sweetened beverages and weight gain: a systematic review. Am J Clin Nutr.

[16] Malik VS, Popkin BM, Bray GA, Despres JP, Willett WC, Hu FB (2010). Sugar-sweetened beverages and risk of metabolic syndrome and type 2 diabetes: a meta-analysis. Diabetes Care.

[17] Bray GA (2012). Fructose and risk of cardiometabolic disease. Curr Atheroscler Rep.

[18] DiNicolantonio JJ, Lucan SC (2014). The wrong white crystals: not salt but sugar as aetiological in hypertension and cardiometabolic disease. Open Heart.

[19] Feig DI, Soletsky B, Johnson R (2008). Effect of allopurinol on blood pressure of adolescents with newly diagnosed essential hypertension: a randomized trial. JAMA.

[20] Nguyen S, Choi H, Lustig R, Hsu C (2009). Sugar-sweetened beverages, serum uric acid, and blood pressure in adolescents. J Pediatr.

[21] Marckmann P (2000). Dietary treatment of thrombogenic disorders related to the metabolic syndrome. Br J Nutr.

[22] Bray GA, Popkin BM (2013). Calorie-sweetened beverages and fructose: what have we learned 10 years later. Pediatr Obes.

[23] DiNicolantonio JJ, O’Keefe JH, Lucan SC (2015). Added fructose: a principal driver of type 2 diabetes mellitus and its consequences. Mayo Clin Proc.

[24] Basu S, Yoffe P, Hills N, Lustig RH (2013). The relationship of sugar to population-level diabetes prevalence: an econometric analysis of repeated cross-sectional data. PLoS ONE.

[25] Goran MI, Ulijaszek SJ, Ventura EE (2013). High fructose corn syrup and diabetes prevalence: a global perspective. Glob Public Health.

[26] World Health Organization (2015) Guideline: sugars intake for adults and children. World Health Organization, Geneva. http://who.int/nutrition/publications/guidelines/sugars_intake/en/25905159

[27] SACN Carbohydrates and Health Report (2015) Scientific Advisory Committee on Nutrition. https://www.gov.uk/government/publications/sacn-carbohydrates-and-health-report. Accessed 9 Dec 2015

[28] USDA (2015) Scientific report of the 2015 Dietary Guidelines Advisory Committee, Advisory Report to the Secretary of Health and Human Services and the Secretary of Agriculture

[29] Food Labeling: Revision of the Nutrition and Supplement Facts Labels; supplemental proposed rule to solicit comment on limited additional provisions. A proposed rule by the food and drug administration on 07/27/2015. https://www.federalregister.gov/articles/2015/07/27/2015-17928/food-labeling-revision-of-the-nutrition-and-supplement-facts-labels-supplemental-proposed-rule-to

[30] Agostoni C, Bresson JL, Fairweather-Tait S (2011). Scientific opinion on the substantiation of health claims related to fructose and reduction of post-prandial glycaemic responses (ID 558) pursuant to Article 13(1) of Regulation (EC) no 1924/2006. EFSA.

[31] Institute of Medicine (U.S.) (2005) Panel on Macronutrients, Institute of Medicine (U.S.). Standing Committee on the Scientific Evaluation of Dietary Reference Intakes. Dietary reference intakes for energy, carbohydrate, fiber, fat, fatty acids, cholesterol, protein, and amino acids. Chapter 6—dietary carbohydrates: sugars and starches. National Academies Press, Washington

[32] Tappy L, Le KA (2010). Metabolic effects of fructose and the worldwide increase in obesity. Physiol Rev.

[33] Johnson RJ, Segal MS, Sautin Y, Nakagawa T, Feig DI, Kang DH (2007). Potential role of sugar (fructose) in the epidemic of hypertension, obesity and the metabolic syndrome, diabetes, kidney disease, and cardiovascular disease. Am J Clin Nutr.

[34] Sun SZ, Empie MW (2012). Fructose metabolism in humans—what isotopic tracer studies tell us. Nutr Metab.

[35] Hellerstein MK, Schwarz JM, Neese RA (1996). Regulation of hepatic de novo lipogenesis in humans. Annu Rev Nutr.

[36] Elliott SS, Keim NL, Stern JS, Teff K, Havel PJ (2002). Fructose, weight gain, and the insulin resistance syndrome. Am J Clin Nutr.

[37] Stanhope KL, Schwarz JM, Keim NL, Griffen SC, Bremer AA, Graham JL, Hatcher B (2009). Consuming fructose-sweetened, not glucose-sweetened, beverages increases visceral adiposity and lipids and decreases insulin sensitivity in overweight/obese humans. J Clin Invest.

[38] Lustig RH (2010). Fructose: metabolic, hedonic, and societal parallels with ethanol. J Am Diet Assoc.

[39] Bray GA, Nielsen SJ, Popkin BM (2004). Consumption of high-fructose corn syrup in beverages may play a role in the epidemic of obesity. Am J Clin Nutr.

[40] White J (2008). Straight talk about high-fructose corn syrup. What it is and what it ain’t. Am J Clin Nutr.

[41] Melanson KJ, Zukley L, Lowndes J, Nguyen V, Angelopoulos TJ, Rippe JM (2007). Effects of high-fructose corn syrup and sucrose consumption on circulating glucose, insulin, leptin, and ghrelin and on appetite in normal-weight women. Nutrition.

[42] Zukley L, Lowndes J, Nguyen V, Brosnahan J, Summers A, Melanson KJ, Angelopoulos TJ, Rippe JM (2007). Consumption of beverages sweetened with high fructose corn syrup and sucrose produce similar levels of glucose, leptin, insulin and ghrelin in obese females. FASEB J.

[43] Yu Z, Lowndes J, Rippe J (2013). High-fructose corn syrup and sucrose have equivalent effects on energy-regulating hormones at normal human consumption levels. Nutr Res.

[44] Soenen S, Westerterp-Plantenga MS (2007). No differences in satiety or energy intake after high-fructose corn syrup, sucrose, or milk preloads. Am J Clin Nutr.

[45] Stanhope KL, Havel PJ (2008). Endocrine and metabolic effects of consuming beverages sweetened with fructose, glucose, sucrose, or high-fructose corn syrup. Am J Clin Nutr.

[46] American Medical Association. Report of the council on science and public health [cited December 9, 2015]. http://www.ama-assn.org/ama1/pub/upload/mm/467/csaph12a07.doc

[47] Fitch C, Keim KS (2012). Position of the academy of nutrition and dietetics: use of nutritive and nonnutritive sweeteners. J Acad Nutr Diet.

[48] Kaiser KA, Shikany JM, Keating KD, Allison DB (2013). Will reducing sugar sweetened beverage consumption reduce obesity? Evidence supporting conjecture is strong, but evidence when testing effect is weak. Obes Rev.

[49] Te Morenga L, Mallard S, Mann J (2013). Dietary sugars and body weight: systematic review and meta-analysis of randomized controlled trials and cohort studies. BMJ.

[50] Malik VS, Pan A, Willett WC, Hu FB (2013). Sugar-sweetened beverages and weight gain in children and adults: a systematic review and meta-analysis. Am J Clin Nutr.

[51] US Department of Agriculture, Economics Research Service 2013. Calories: average daily per capita calories from the US food supply, adjusted for spoilage and other waste. Loss-Adjusted Food Availability Data

[52] Eshak ES, Iso H, Kokubo Y, Saito I, Yamagishi K, Inoue M, Tsugane S (2012). Soft drink intake in relation to incident ischemic heart disease, stroke, and stroke subtypes in Japanese men and women: the Japan Public Health Centre—based study cohort I. Am J Clin Nutr.

[53] de Koning L, Malik VS, Kellogg MD, Rim EB, Willett WC, Hu FB (2012). Sweetened beverage consumption, incident coronary heart disease and biomarkers of risk in men. Circulation.

[54] Fung T, Malik V, Rexrode K, Manson JE, Willett WC, Hu FB (2009). Sweetened beverage consumption and risk of coronary heart disease in women. Am J Clin Nutr.

[55] Te Morenga LA, Howatson AJ, Jones RM, Mann J (2014). Dietary sugars and cardiometabolic risk: systematic review and meta-analyses of randomized controlled trials of the effects on blood pressure and lipids. Am J Clin Nutr.

[56] Brown CM, Dulloo AG, Yepuri G, Montani JP (2008). Fructose ingestion acutely elevates blood pressure in healthy young humans. Am J Physiol Regul Integr Comp Physiol.

[57] Raben A, Vasilaras T, Møller A, Astrup A (2002). Sucrose compared with artificial sweeteners: different effects on ad libitum food intake and body weight after 10 wk of supplementation in overweight subjects. Am J Clin Nutr.

[58] Lê K-A, Faeh D, Stettler R, Ith M, Kreis R, Vermathen P, Boesch C, Ravussin E, Tappy L (2006). A 4-wk high-fructose diet alters lipid metabolism without affecting insulin sensitivity or ectopic lipids in healthy humans. Am J Clin Nutr.

[59] Maersk M, Belza A, Stødkilde-Jørgensen H, Ringgaard S, Chabanova E, Thomsen H (2012). Sucrose sweetened beverages increase fat storage in the liver, muscle, and visceral fat depot: a 6-mo randomized intervention study. Am J Clin Nutr.

[60] Lowndes J, Sinnett S, Grench K, Jordan R, Rippe J (2014). Impact of fructose and fructose containing sugars on indices of cardiometabolic health when consumed at typical levels. Circulation.

[61] Lowndes J, Sinnett S, Yu Z, Rippe J (2014). The effects of fructose containing sugars on weight, body composition and cardiometabolic risk factors when consumed at up to the 90th percentile population consumption level for fructose. Nutrients.

[62] Angelopoulos TJ, Lowndes J, Sinnett S, Rippe JM (2015). Fructose containing sugars do not raise blood pressure or uric acid at normal levels of human consumption. J Clin Hypertens.

[63] Ha V, Sievenpiper JL, de Souza RJ, Chiavaroli L, Wang DD, Cozma AI, Mirrahimi A, Yu ME (2012). Effect of fructose on blood pressure: a systematic review and meta-analysis of controlled feeding trials. Hypertension.

[64] Gross LS, Li L, Ford ES, Liu S (2004). Increased consumption of refined carbohydrates and the epidemic of type 2 diabetes in the United States: an ecologic assessment. Am J Clin Nutr.

[65] Dhingra R, Sullivan L, Jacques PF, Wang TJ, Fox CS, Meigs JB, D’Agostino RB (2007). Soft drink consumption and risk of developing cardiometabolic risk factors and the metabolic syndrome in middle-aged adults in the community. Circulation.

[66] Miller M, Stone N, Ballantye C, Vittner V, Criqui MH, Ginsberg HN, Goldberg AC (2011). Triglycerides and cardiovascular disease: a scientific statement from the American Heart Association. Circulation.

[67] Chiavaroli L, Mirrahimi A, De Souza RJ, Cozma A, Ha V, Wang DD, Yu ME (2012). Does fructose consumption elicit a dose-response effect on fasting triglycerides? A systematic review and meta-regression of controlled feeding trials. Can J Diabetes.

[68] Wang DD, Sievenpiper JL, de Souza RJ, Cozma AI, Chiavaroli L, Ha V, Mirrahimi A (2014). Effect of fructose on postprandial triglycerides: a systematic review and meta analysis of controlled feeding trials. Atherosclerosis.

[69] Zhang Y, An T, Zhang R, Zhou Q, Huang Y, Zhang J (2013). Very high fructose intake increases serum LDL-cholesterol and total cholesterol: a meta-analysis of controlled feeding trials. J Nutr.

[70] Egli L, Lecoultre V, Theytaz F, Campos V, Hodson L, Schneiter P, Mittendorfer B (2013). Exercise prevents fructose-induced hypertriglyceridemia in healthy young subjects. Diabetes.

[71] Lowndes J, Sinnett S, Pardo S, Nguyen V, Melanson K, Yu Z, Lowther B, Rippe J (2014). The effect of normally consumed amounts of sucrose or high fructose corn syrup on body composition and related parameters in overweight/obese subjects. Nutrients.

[72] Johnson RK, Appel LJ, Brands M, Howard BV, Lefevre M, Lustig RH, Sacks F, Steffen LM, Wylie-Rosett F, on behalf of the American Heart Association Nutrition Committee of the Council on Nutrition, Physical Activity and Metabolism and the Council on Epidemiology and Prevention (2009). Dietary Sugars Intake and Cardiovascular Health, A Scientific Statement from the American Heart Association. Circulation.

[73] van Buul VJ, Tappy L, Brouns FJ (2014). Misconceptions about fructose-containing sugars and their role in the obesity epidemic. Nutr Res Rev.

[74] Barclay AW, Brand-Miller J (2011) The Australian paradox: a substantial decline in sugars intake over the same timeframe that overweight and obesity have increased. Nutrients 3:491–504. Erratum in: Nutrients 2014;6:663–634 10.3390/nu3040491 PMC3257688 22254107

[75] Welsh JA, Sharma AJ, Grellinger L, Vos MB (2011). Consumption of added sugars is decreasing in the united states. Am J Clin Nutr.

[76] Janket SJ, Manson JE, Sesso H, Buring JE, Liu S (2003). A prospective study of sugar intake and risk of type 2 diabetes in women. Diabetes Care.

[77] de Koning L, Malik VS, Kellogg MD, Rimm EB, Willett WC, Hu FB (2012). Sweetened beverage consumption, incident coronary heart disease and biomarkers of risk in men. Circulation.

[78] Hodge AM, English DR, O’Dea K, Giles DD (2004). Glycemic index and dietary fiber and the risk of type 2 diabetes. Diabetes Care.

[79] Colditz GA, Manson JE, Stampfer MJ, Rosner B, Willett WC, Speizer FE (1992). Diet and risk of clinical diabetes in women. Am J Clin Nutr.

[80] Meyer KA, Kushi LH, Jacobs DR, Slavin J, Sellers TA, Folson AR (2000). Carbohydrates, dietary fiber, and incident type 2 diabetes in older women. Am J Clin Nutr.

[81] Cozma AI, Sievenpiper JL, de Souza RJ, Chiavaroli L, Ha V, Wang DD, Mirrahimi A (2012). Effect of fructose on glycemic control in diabetes: a systematic review and meta-analysis of controlled feeding trials. Diabetes Care.

[82] Teff KL, Elliott S, Tschop M, Kieffer TJ, Rader D, Heiman M, Townsend RR (2004). Dietary fructose reduces circulating insulin and leptin, attenuates postprandial suppression of ghrelin, and increases triglycerides in women. J Clin Endocrinol Metab.

[83] Aeberli I, Gerber PA, Hochuli M, Kohler S, Haile SR, Gouni-Berthold I, Berthold HK (2011). Low to moderate sugar sweetened beverage consumption impairs glucose and lipid metabolism and promotes inflammation in healthy young men: a randomized controlled trial. Am J Clin Nutr.

[84] Aeberli I, Hochuli M, Gerber PA, Sze L, Murer SB, Tappy L, Spinas GA, Berneis K (2013). Moderate amounts of fructose consumption impair insulin sensitivity in healthy young men: a randomized controlled trial. Diabetes Care.

[85] Stanhope KL, Griffen SC, Bremer AA, Vink RG, Schaefer EJ, Nakajima K, Schwarz J-M (2011). Metabolic responses to prolonged consumption of glucose- and fructose-sweetened beverages are not associated with postprandial or 24-h glucose and insulin excursions. Am J Clin Nutr.

[86] Beck-Nielsen H, Pedersen O, Lindskov HO (1980). Impaired cellular insulin binding and insulin sensitivity induced by high-fructose feeding in normal subjects. Am J Clin Nutr.

[87] Shulman GI (2014). Ectopic fat in insulin resistance, dyslipidemia, and cardiometabolic disease. N Engl J Med.

[88] Lowndes J, Sinnett S, Rippe J (2015). No effect of added sugar consumed at median american intake level on glucose tolerance or insulin resistance. Nutrients.

[89] Matsuda M, Liu Y, Mahankali S, Pu Y, Mahankali A, Wang J, DeFronzo RA, Fox PT, Gao JH (1999). Altered hypothalamic function in response to glucose ingestion in obese humans. Diabetes.

[90] Johnston RD, Stephenson MC, Crossland H, Cordon SM, Palcidi E, Cox EF, Taylor MA, Aithal GP, Macdonald IA (2013). No difference between high-fructose and high-glucose diets on liver triacylglycerol or biochemistry in healthy overweight men. Gastroenterology.

[91] Ouyang X, Cirillo P, Sautin Y, McCall S, Bruchette JL, Diehl AM, Johnson RJ, Abdelmalek MF (2008). Fructose consumption as a risk factor for non-alcoholic fatty liver disease. J Hepatol.

[92] Thuy S, Ladurner R, Volynets V, Wagner S, Strahl S, Konigsrainer A, Maier KP (2008). Nonalcoholic fatty liver disease in humans is associated with increased plasma endotoxin and plasminogen activator inhibitor 1 concentrations and with fructose intake. J Nutr.

[93] Parks EJ, Skokan LE, Timlin MT, Dingfelder CS (2008). Dietary sugars stimulate fatty acid synthesis in adults. J Nutr.

[94] Lê KA, Ith M, Kreis R, Faeh D, Bortolotii M, Tran C, Boesch C, Tappy L (2009). Fructose overconsumption causes dyslipidemia and ectopic lipid deposition in healthy subjects with and without a family history of type 2 diabetes. Am J Clin Nutr.

[95] Bravo S, Lowndes J, Sinnett S, Yu Z, Rippe J (2013). Consumption of sucrose and high-fructose corn syrup does not increase liver fat or ectopic fat deposition in muscles. Appl Physiol Nutr Metab.

[96] Funari VA, Herrera VL, Freeman D, Tolan DR (2005). Genes required for fructose metabolism are expressed in Purkinje cells in the cerebellum. Brain Res Mol Brain Res.

[97] Lindqvist A, Mohapel P, Bouter B, Frielingsdorf H, Pizzo D, Brundin P, Erlanson-Albertsson C (2006). High-fat diet impairs hippocampal neurogenesis in male rats. Eur J Neurol.

[98] Smeets PA, de Graaf C, Stafleu A, van Osch MJ, van der Grond J (2005). Functional magnetic resonance imaging of human hypothalamic responses to sweet taste and calories. Am J Clin Nutr.

[99] Page KA, Luo S, Romero A, Adam T, Hu HH (2012) Fructose compared to glucose ingestion preferentially activates brain reward regions in response to high-calorie food cues in young, obese Hispanic females. Endocrinol Rev 33:1666

[100] Purnell JQ, Klopfenstein BA, Stevens AA, Havel PJ, Adams SH, Dunn TN (2011). Brain functional magnetic resonance imaging response to glucose and fructose infusions in humans. Diabetes Obes Metab.

[101] Benton D (2010). The plausibility of sugar addiction and its role in obesity and eating disorders. Clin Nutr.

[102] Ziauddeen H, Farooqi I, Fletcher P (2012). Obesity and the brain: how convincing is the addiction model?. Nat Rev Neurosci.

[103] Corwin LW, Hayes JE, Rippe JM (2014). Are the sugars addictive? Perspectives for practitioners. Fructose, high fructose corn syrup, sucrose and health.

[104] Lowndes J, Yu Z, Rippe JM (2015) No dose response relationship in the effects of commonly consumed sugars on insulin sensitivity across a range of typical human consumption levels. Experimental Biology C264, 591.11. FASEB J 29

